# Geographic pattern of antibiotic resistance genes in the metagenomes of the giant panda

**DOI:** 10.1111/1751-7915.13655

**Published:** 2020-08-18

**Authors:** Ting Hu, Qinlong Dai, Hua Chen, Zheng Zhang, Qiang Dai, Xiaodong Gu, Xuyu Yang, Zhisong Yang, Lifeng Zhu

**Affiliations:** ^1^ College of Life Sciences Nanjing Normal University Nanjing 210046 China; ^2^ Sichan Liziping National Nature Reserve Shimian China; ^3^ Shimian Research Center of Giant Panda Small Population Conservation and Rejuvenation Shimian China; ^4^ Mingke Biotechnology Co., Ltd. Hangzhou China; ^5^ Chengdu Institute of Biology Chinese Academy of Sciences Chengdu China; ^6^ Sichuan Station of Wildlife Survey and Management Chengdu 610082 China; ^7^ Key Laboratory of Southwest China Wildlife Resources Conservation (Ministry of Education) China West Normal University Nanchong 637002 China

## Abstract

The rise in infections by antibiotic‐resistant bacteria poses a serious public health problem worldwide. The gut microbiome of animals is a reservoir for antibiotic resistance genes (ARGs). However, the correlation between the gut microbiome of wild animals and ARGs remains controversial. Here, based on the metagenomes of giant pandas (including three wild populations from the Qinling, Qionglai and Xiaoxiangling Mountains, and two major captive populations from Yaan and Chengdu), we investigated the potential correlation between the constitution of the gut microbiome and the composition of ARGs across the different geographic locations and living environments. We found that the types of ARGs were correlated with gut microbiome composition. The NMDS cluster analysis using Jaccard distance of the ARGs composition of the gut microbiome of wild giant pandas displayed a difference based on geographic location. Captivity also had an effect on the differences in ARGs composition. Furthermore, we found that the Qinling population exhibited profound dissimilarities of both gut microbiome composition and ARGs (the highest proportion of *Clostridium* and vancomycin resistance genes) when compared to the other wild and captive populations studies, which was supported by previous giant panda whole‐genome sequencing analysis. In this study, we provide an example of a potential consensus pattern regarding host population genetics, symbiotic gut microbiome and ARGs. We revealed that habitat isolation impacts the ARG structure in the gut microbiome of mammals. Therefore, the difference in ARG composition between giant panda populations will provide some basic information for their conservation and management, especially for captive populations.

## Graphical abstract

The wild giant panda in their habitat in Liziping National Nature Reserve, Shimian. The copywight belongs to Liziping National Nature Reserve, Shimian.

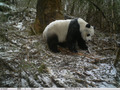

## Introduction

Worldwide, the rise in infections by antibiotic‐resistant bacteria poses a serious public health problem (Stewart and Costerton, 2001; Ventola, 2015). Host diet and phylogeny are two main factors influencing animal gut microbiome composition and function (Ley *et al*., 2008; Muegge *et al*., 2011), and the gut microbiome of animals plays an important role in host immunity, development and health (Kinross *et al*., 2008; Spor *et al*., 2011; Wei *et al*., 2019). However, their gut microbiome is also considered to be a reservoir for antibiotic resistance genes (ARGs; Sommer *et al*., 2009; Looft *et al*., 2012; Zhou *et al*., 2012; Hu *et al*., 2013; Laxminarayan *et al*., 2013; Allen, 2014; Fitzpatrick and Walsh, 2016). Previous studies have revealed that the environment or habitat has a profound effect on the types of ARGs present in the microbiome (Forsberg *et al*., 2014; Pal *et al*., 2016). For example, different habitats (e.g. different human body sites, water, and soils) harbour different symbiotic microbiome communities and have a different composition of ARGs (Pal *et al*., 2016). In humans, the gut microbiome of people from different countries also shows a difference in ARGs composition, to some extent (Feng *et al*., 2018). Therefore, these findings indicate a potential correlation between the structure of the gut microbiome and the composition of ARGs. Moreover, the ARG structure in the gut microbiome within the same species may differ based on a geographic pattern. However, this hypothesis needs further supporting evidence.

**Fig. 1:**
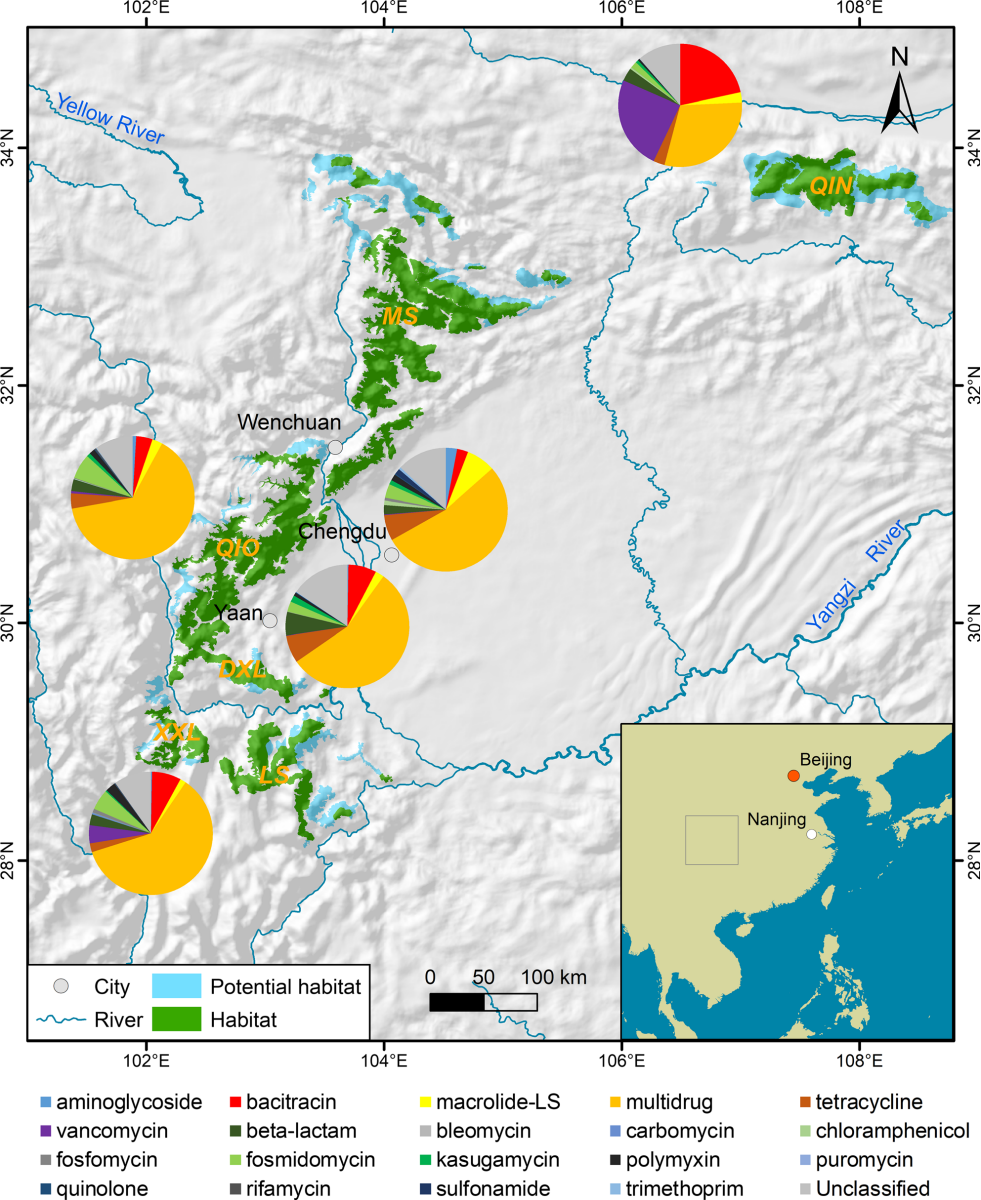
The wild giant panda habitats and antibiotic resistance genes (ARGs) structure (mean abundance). Six wild mountain giant panda populations: Qinling Mountains population (QIN), Minshan Mountains population (MS), Qionglai Mountains population (QIO), Daxiangling Mountains population (DXL), Xiaoxiangling Mountains population (XXL) and Liangshan Mountains population (LS). Two captive populations: Chengdu Breeding Center (CD) and the Giant Panda Research Center in Yaan (Yaan). In this study, the metagenomes from five populations (three wild populations: QIN, QIO and XXL; two captive populations: CD and Yaan) were analysed. Macrolide‐LS: macrolide–lincosamide–streptogramin.

The giant panda, which belongs to the Carnivora order, lives in six mountain regions in China (Fig. 1), including the Qinling Mountains, Minshan Mountains, Qionglai Mountains, Daxiangling Mountains, Xiaoxiangling Mountains and Liangshan Mountains (Schaller *et al*., 1985). Additionally, there are several captive populations, such as the Wolong Research Center (located in Wolong and Yaan) and Chengdu Breeding Center (located in Chengdu). The giant panda, along with the sympatric species the red panda, is a bamboo‐eating panda (herbivorous carnivorans; Schaller *et al*., 1985; Wei *et al*., 2000). There are two common findings in the giant panda gut microbiomes. First, previous studies have found a difference in the gut microbiome community between wild and captive populations of giant pandas (Zhu *et al*., 2011; Wei *et al*., 2015; Xue *et al*., 2015; Guo *et al*., 2019; Yao *et al*., 2019). Second, the gut microbiome of wild pandas in the Shaanxi region (only one wild population in the Qinling Mountains) harbours a high proportion of *Clostridiaceae* (Zhu *et al*., 2011; Wu *et al*., 2017). Considering the potential correlation between the community of the gut microbiome and the composition of ARGs, there are no large‐scale meta‐analyses of the gut microbiome of giant pandas and the composition ARGs across wild and captive populations from different geographic areas.

Here, based on the published metagenomes of giant pandas [including three wild populations from the Qinling (Wu *et al*., 2017), Qionglai (Guo *et al*., 2019) and Xiaoxiangling Mountains (Zhu *et al*., 2018a), and two major captive populations from Wolong (Guo *et al*., 2019) and Chengdu (Zhang *et al*., 2018)] by both our group and other groups, we mainly aimed to investigate the potential correlation between the structure of the gut microbiome and the composition of ARGs across different geographic locations and living environments. In addition, we also integrated other published Carnivora metagenomes (including meat‐eating carnivorans (Zhu *et al*., 2018b) and omnivorous carnivorans (Guo *et al*., 2018; Zhu *et al*., 2018b)) to investigate the effect of diet on the ARGs in the gut microbiome.

## Results and discussion

In this study, we analysed the metagenomes of 96 mammals: 19 meat‐eating carnivorans (CA), ten omnivorous carnivorans (OC), 55 bamboo‐eating carnivorans [49 giant panda samples: nine from the Qinling Mountains (QIN, wild), seven from the Qionglai Mountains (QIO, wild), 16 from the Xiaoxiangling Mountains (XXL, wild), seven from the Chengdu Breeding Center (CD, captive), ten from the Yaan research base of the Wolong Research Center (Yaan, captive) and six from red pandas in the Xiaoxiangling Mountains (wild)], and 12 herbivores (HE; Table S1).

### The dissimilarity in both the gut microbiome community and ARG composition among the four diet groups

**Fig. 2:**
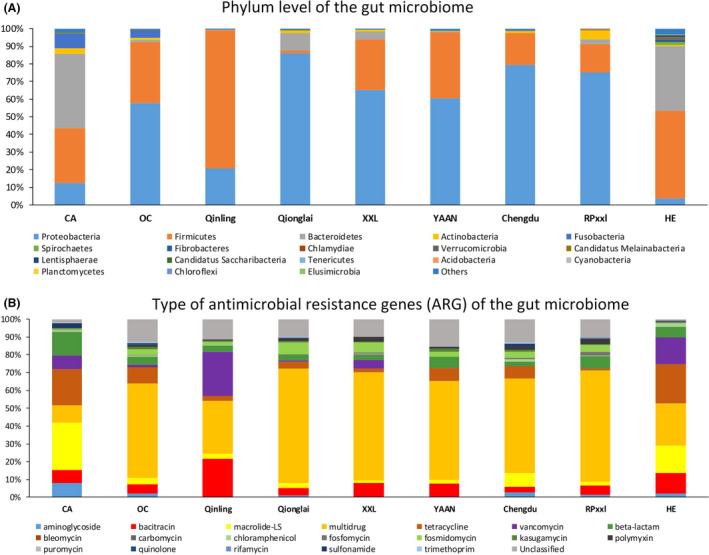
The gut microbiome composition and ARG patterns in giant pandas. A. The phylum level of the gut microbiome identified in the 96 metagenomes included in the study. B. The type of ARGs identified in the gut microbiome. CA, meat‐eating carnivorans. OC, omnivorous carnivorans. HE, herbivores. Qinling, wild Qinling giant panda populations. Qionglai, wild Qionglai giant panda population. XXL, wild Xiaoxiangling giant panda population. RPxxl, wild Xiaoxiangling red panda population. Yaan, the captive Yaan giant panda population. Chengdu, the captive Chengdu giant panda population. Macrolide‐LS: macrolide–lincosamide–streptogramin.

**Fig. 3:**
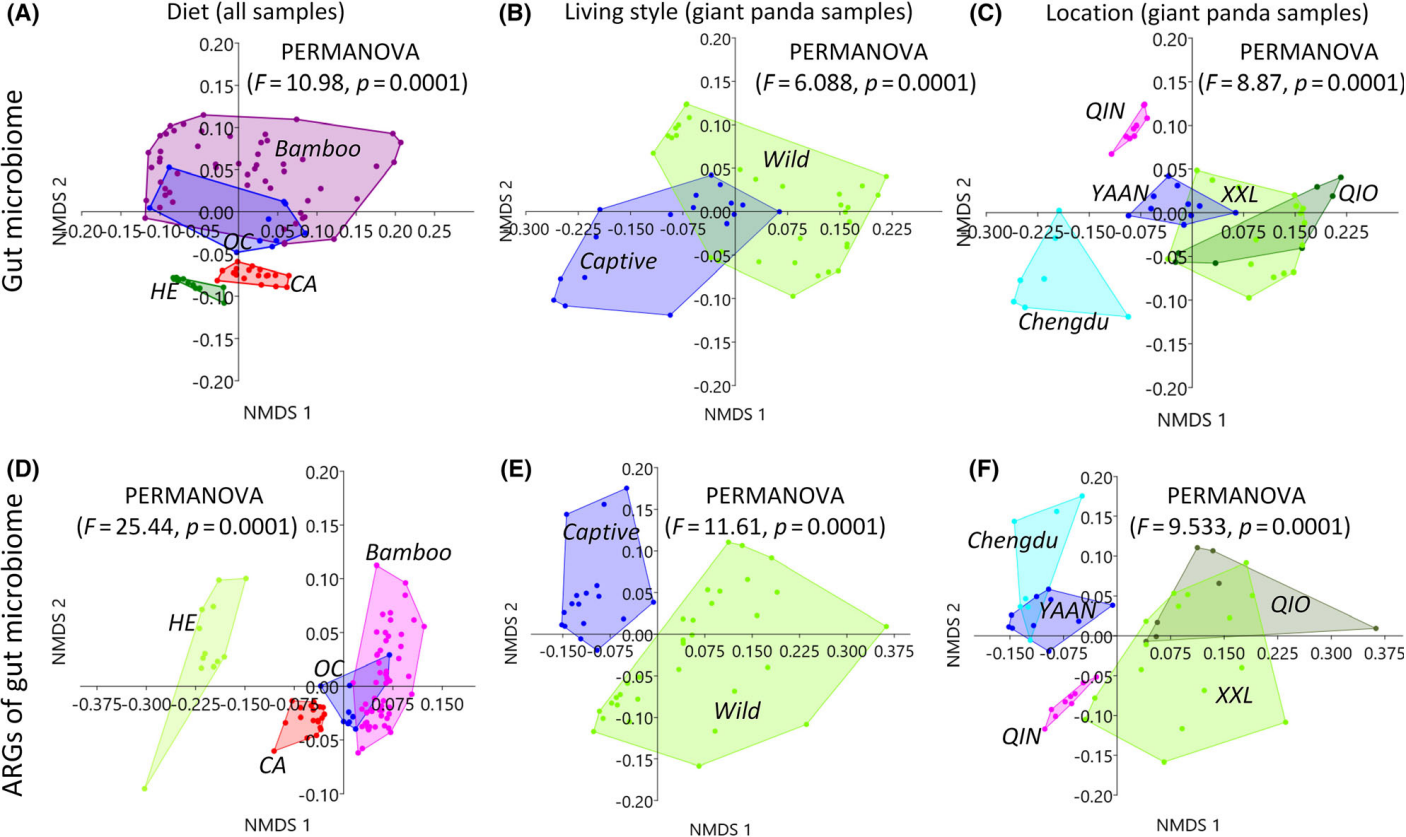
The differences in metagenome between groups were generated via the non‐metric multidimensional scaling (NMDS) method using the Jaccard distance. A. The effect of diet on the similarities in gut microbiome communities between four groups (including 96 metagenomes, all samples used in this study) using the Jaccard distance of gut microbial genera abundance. B. The effect of living environment (e.g. captivity) on the similarities in gut microbiome communities between two groups (only including giant panda metagenomes) using the Jaccard distance of gut microbial genera abundance. C. The effect of geographic location on the similarities in gut microbiome communities between five groups (only including giant panda metagenomes) using the Jaccard distance of gut microbial genera abundance. D. The effect of diet on the similarities in ARG composition between four groups (including 96 metagenomes, all samples used in this study) using the Jaccard distance of the ARG subtype abundance. E. The effect of living environment (e.g. captivity) on the similarities in ARG composition between two groups (only including giant panda metagenomes) using the Jaccard distance of the ARG subtype abundance. F. The effect of geographic location on the similarities in ARG composition between five groups (only including giant panda metagenomes) using the Jaccard distance of the ARG subtype abundance. CA, meat‐eating carnivorans. OC, omnivorous carnivorans. Bamboo, bamboo‐eating carnivorans (giant panda and red panda). HE, herbivores. Captive, the captive giant pandas including the Chengdu and Yaan populations. Wild, wild giant pandas including the Qinling, Qionglai, and Xiaoxiangling populations. QIN, the wild Qinling giant panda population. QIO, the wild Qionglai population. XXL, the wild Xiaoxiangling population. Yaan, the captive Yaan population. Chengdu, the captive Chengdu population.

Our results showed a significant difference in both the gut microbiome community and ARG composition between the four diet groups (using all samples; Figs 2 and 3A,D, and Jaccard distance: PERMANOVA, *P* = 0.0001), and this difference was larger in regard to the ARG composition when compared to that in the gut microbiome community [PERMANOVA: *F* value (25.44) of ARGs > *F* value (10.98) of gut microbiome]. For example, at the phylum level (Fig. 2A), the dominant populations in the gut microbiome of CA included Bacteroidetes, Firmicutes, Proteobacteria and Fusobacteria. The main phyla in OC included Proteobacteria and Firmicutes, while the dominant populations in the gut microbiome of HE included Firmicutes and Bacteroidetes. Altogether, Proteobacteria and Firmicutes were the dominant populations in giant panda and red panda samples (Fig. 2A). In regard to the types of ARGs (Fig. 2B), the dominant ARGs in the gut microbiome of CA populations included macrolide–lincosamide–streptogramin, tetracycline, and beta‐lactam resistance genes. The main types of ARGs in OC populations included multidrug resistance genes and tetracycline resistance genes, whereas the dominant types of ARGs in HE populations included multidrug resistance genes, as well as macrolide–lincosamide–streptogramin and tetracycline resistance genes. Taken together, the mean abundance of multidrug resistance genes revealed them to be the dominant type of ARGs in giant panda and red panda samples (Fig. 2B). Therefore, combining with the previous findings on the difference in the gut microbiome among different diet mammal groups (e.g. Ley *et al*., 2008; Muegge *et al*., 2011; Zhu *et al*., 2018a), we here revealed the dissimilarity in ARG composition in their gut microbiome.

### The high proportion of tetracycline and macrolides resistance genes in captive mammals

**Fig. 4:**
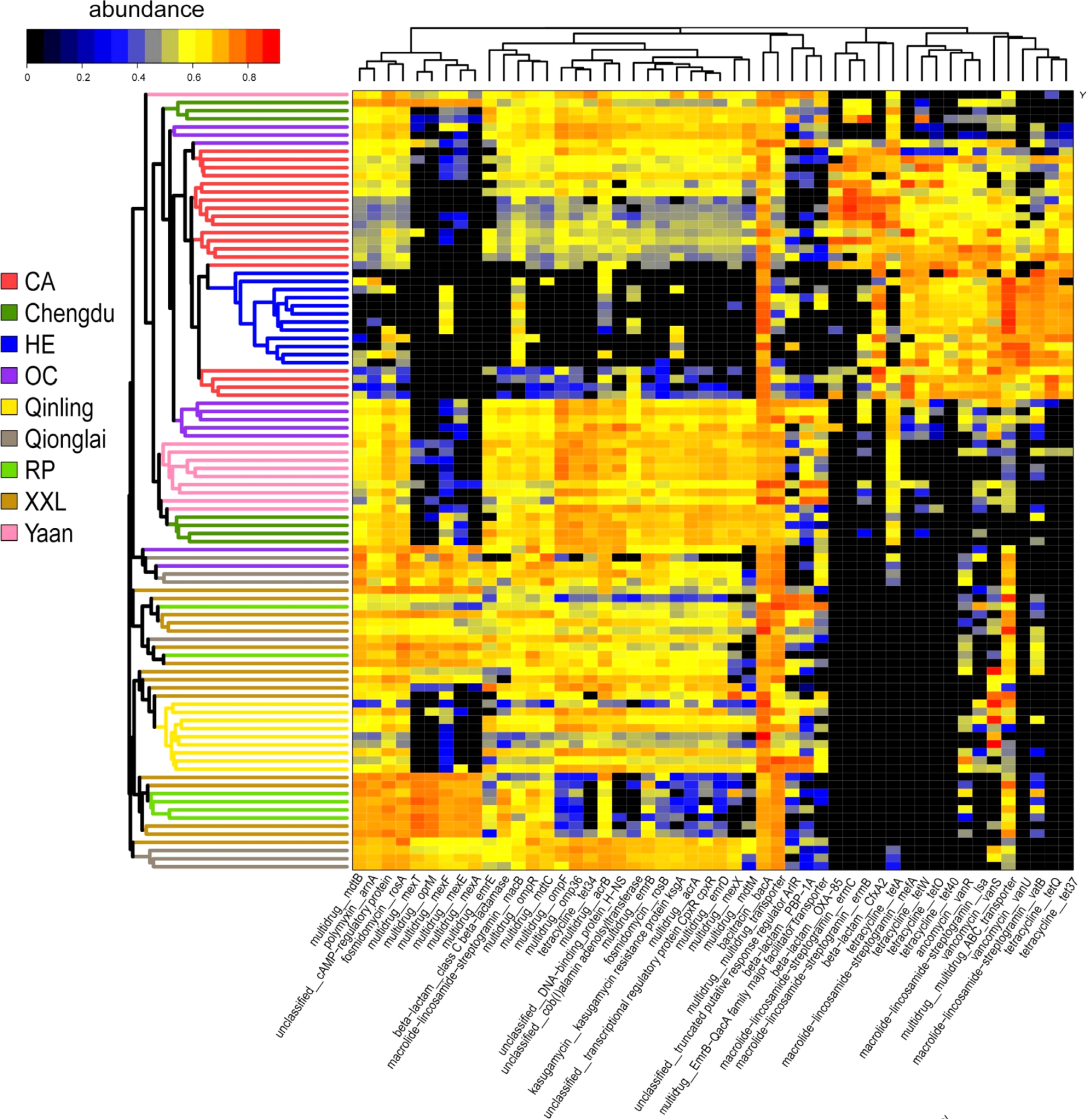
The heatmap of the top 50 ARG subtypes in the 96 metagenomes studied. The left tree is a neighbour‐joining tree constructed using the Jaccard distance of the ARG subtype abundance, with each colour representing one group. In the main figure, each row represents one metagenome. Each column represents one ARG subtype. In order to display the heatmap clearly, the abundance was transformed from the original abundance using the following formula: lg^(original abundance x 1000)^.

**Fig. 5:**
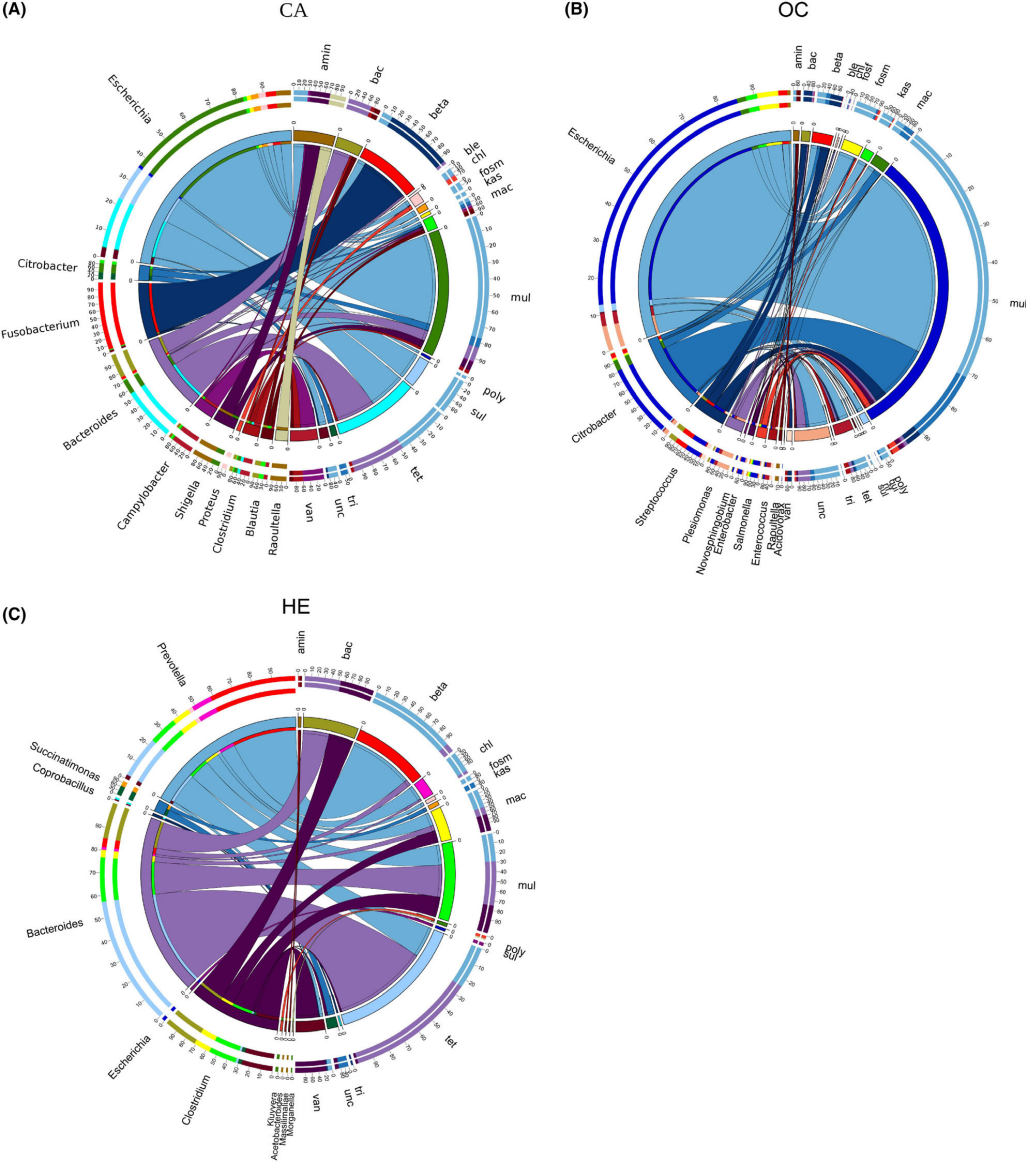
The distributions of ARG types and their abundance in the total annotated ARGs in the metagenomes [visualized by Circos (Krzywinski *et al*., 2009)]. The length of the bars on the outer ring represents the percentage of gut microbiome groups (genera level) for each ARG type. Each gut microbiome genus was represented by a specific ribbon colour and the width of each ribbon shows the abundance of each genus in the ARG type. A. CA (meat‐eating carnivorans). B. OC (omnivorous carnivorans). C. HE (herbivores). amin, aminoglycoside. bac, bacitracin. beta, beta‐lactam. ble, bleomycin. chl, chloramphenicol. fosm, fosmidomycin. kas, kasugamycin. mac, macrolide–lincosamide–streptogramin. mul, multidrug. poly, polymyxin. sul, sulfonamide. tet, tetracycline. tri, trimethoprim. unc, unclassified. van, vancomycin.

We further discovered the high proportion of tetracycline and macrolides resistance genes in captive mammals in this study, especially in CA and HE groups (Figs 2B and 4). Tetracycline and macrolides are widely used to treat bacterial infections in various body systems (e.g. the skin, intestines and respiratory tract; Klein and Cunha, 1995; Anadon and Reevejohnson, 1999). They have been widely applied in many species (e.g. dogs, cats, cattle, sheep, swine, turkeys and chickens) for veterinary use in many places, such as zoos and farms, as well as for domestic animals (Stuart and Smith, 1992; Anadon and Reevejohnson, 1999; Winckler and Grafe, 2001; Li *et al*., 2018). As such, this may explain the higher proportion of tetracycline and macrolides resistance genes in captive mammals. However, the putative gut microbiome genera, which harboured tetracycline and macrolides resistance genes, were found to differ. In CA, the tetracycline resistance genes were mainly identified in *Bacteroides* and *Escherichia*, in OC and captive pandas mainly in *Escherichia*, and in HE mainly in *Bacteroides* and *Prevotella* (Fig. 5, Fig. S1). In CA, macrolide–lincosamide–streptogramin resistance genes were mainly in *Escherichia*, *Blautia* and *Citrobacter*, and in HE mainly in *Bacteroides*, *Prevotella* and *Clostridium* (Fig. 5, Figs S1‐S2). Therefore, the consensus pattern on the ARG composition in the captive mammal gut microbiome might be associated with the used antibiotics in the captivity, but the putative gut bacteria sources might be different given that the effect by the different host phylogeny position.

### The captivity potentially resulting in the divergence in the ARG composition of the giant panda gut microbiome

Based on the meta‐analysis of the giant panda metagenome data, we confirmed the previous findings (mostly using the 16S rRNA data) on the difference in the gut microbiome composition (Wei *et al*., 2015; Xue *et al*., 2015; Guo *et al*., 2019; Yao *et al*., 2019). At the genus level, the dominant populations in the gut microbiome differed among giant panda populations (Table S2). The mean abundance of *Escherichia* (Proteobacteria) was highest in captive giant panda populations (0.34 ± 0.19 in Yaan, and 0.28 ± 0.21 in CD). In wild giant pandas, the mean abundance of *Clostridium* (Firmicutes) was highest in the QIN population (0.42 ± 0.35), and the mean abundance of *Pseudomonas* (Proteobacteria) was highest in non‐QIN populations (0.22 ± 0.19 in Qionglai, 0.44 ± 0.41 in XXL). Additionally, the mean abundance of *Pseudomonas* (Proteobacteria) was also highest in the XXL population of wild red pandas (0.61 ± 0.38). However, the new finding here was the dissimilarity in the ARG composition between the captive and wild giant panda gut microbiome.

**Fig. 6:**
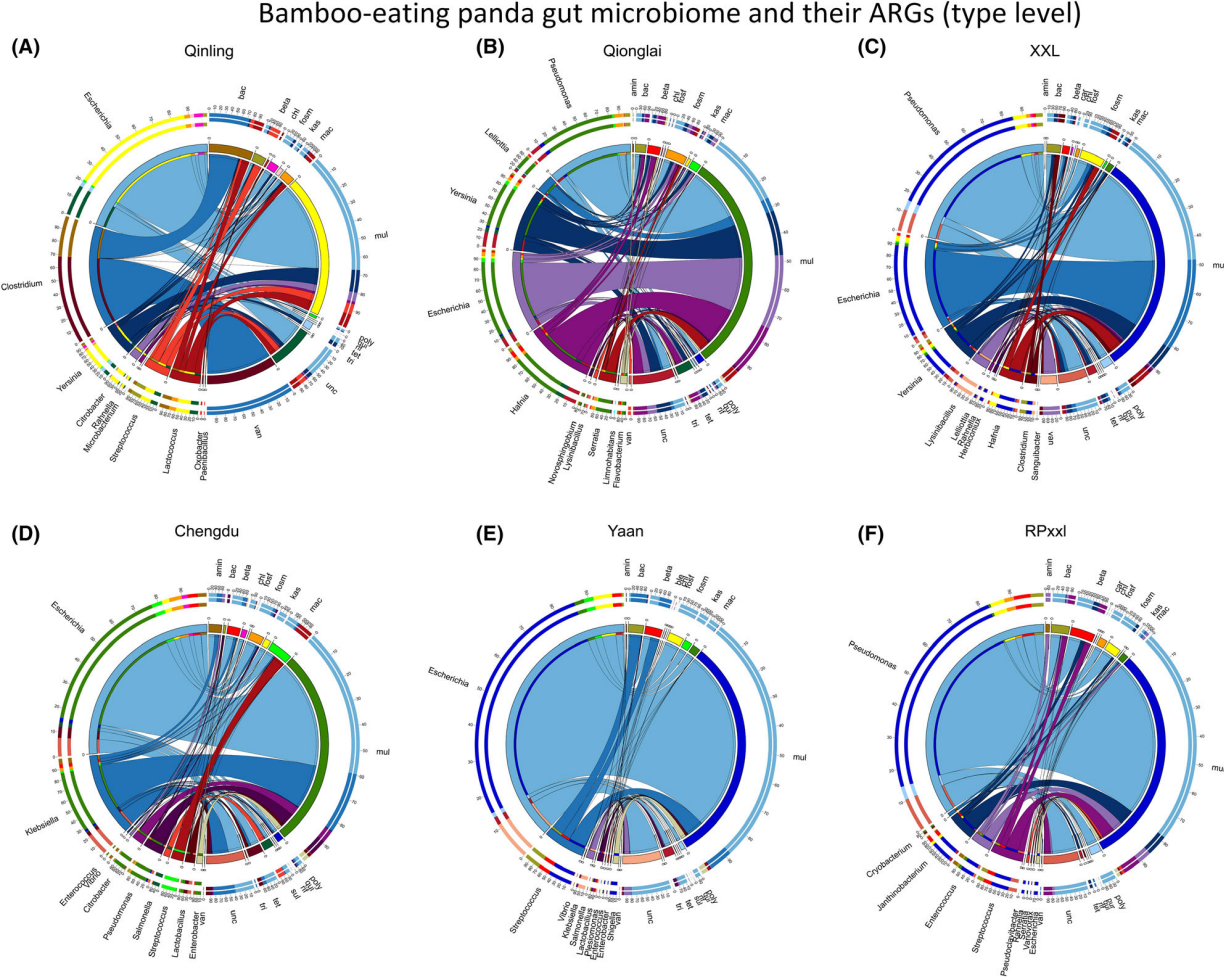
The distribution of ARG types and their abundance in the total annotated ARGs in giant panda and red panda metagenomes [visualized by Circos (Krzywinski *et al*., 2009)]. The length of the bars on the outer ring represents the percentage of gut microbiome groups (genera level) for each ARG type. Each gut microbiome genus was represented by a specific ribbon colour and the width of each ribbon indicates the abundance of each genus in the ARG type. A. Qinling (wild Qinling population). B. Qionglai (wild Qionglai population). C. XXL (wild Xiaoxiangling population). D. Chengdu (captive Chengdu population). E. Yaan (captive Yaan population). F. RPxxl (wild red panda Xiaoxiangling population). amin, aminoglycoside. bac, bacitracin. beta, beta‐lactam. ble, bleomycin. chl, chloramphenicol. fosm, fosmidomycin. kas, kasugamycin. mac, macrolide–lincosamide–streptogramin. mul, multidrug. poly, polymyxin. sul, sulfonamide. tet, tetracycline. tri, trimethoprim. unc, unclassified. van, vancomycin.

By only taking into account the metagenomes of giant pandas, we also found a significant difference in both gut microbiome community and ARG composition between the captive and wild groups (Fig. 3B,E, Jaccard distance, PERMANOVA, *P* = 0.0001). This difference in ARG composition between the groups was larger than that in the gut microbiome community, with two clear clusters for ARGs (Fig. 3E, Captive vs. Wild). For example, the mean abundance of tetracycline resistance genes was higher in captive populations (0.07 ± 0.02 in Yaan and 0.07 ± 0.04 in CD) than that found in wild populations (0.03 ± 0.03 in QIN, 0.04 ± 0.02 in Qionglai, and 0.02 ± 0.02 in XXL). The gut microbiome of captive populations had a higher abundance of several ARG subtypes, including tetracycline_tet34, and multidrug_ompF. In captive CD populations, tetracycline resistance genes were mainly identified in *Escherichia*, multidrug resistance genes in *Escherichia*, *Klebsiella*, *Citrobacter*, and *Pseudomonas*, and macrolide–lincosamide–streptogramin resistance genes in *Streptococcus* (Fig. 6 and Fig. S2). In the captive Yaan giant panda population, tetracycline resistance genes were mainly found in *Escherichia* and *Lactobacillus*, multidrug resistance genes in *Escherichia*, *Streptococcus*, and *Shigella*, and macrolide–lincosamide–streptogramin resistance genes in *Escherichia* (Fig. 6 and Fig. S2). As mentioned above, some antibiotics (e.g. tetracycline, macrolides, gentamicin, azithromycin and cephalosporins) are used in the captive giant panda population, which might have the effect on the ARGs in the gut microbiome and resulted in the divergence with those in the wild giant pandas.

### The geographic divergence in the composition of ARGs in gut microbiome among wild giant panda mountain populations

We then divided the giant panda metagenome samples into five groups based on their geographic locations and found a significant difference in both gut microbiome community and ARG composition (Fig. 3C,F, Jaccard distance, PERMANOVA, *P* = 0.0001). As such, our results revealed a clear divergence between QIN and non‐QIN populations (Qionglai and XXL populations) (Fig. 3C,F). Notably, the QIN population exhibited a unique pattern of ARG types, with the abundance of the vancomycin (0.25 ± 0.29) and bacitracin (0.22 ± 0.26) resistance genes being the highest when compared to the other wild or captive populations (Fig. 2B). In regard to the ARG subtypes (Fig. 6 and Table S3), the mean abundance of vancomycin_vanS (0.21 ± 0.30) and bacitracin_bacA (0.22 ± 0.26) was the highest in the gut microbiome of giant pandas from the QIN population when compared to the other wild or captive populations. In the QIN giant panda population (wild), the vancomycin resistance genes (e.g. vancomycin_vanS) were identified in *Clostridium* (Fig. 6 and Fig. S2). The mean abundance of several ARG subtypes from multidrug resistance genes type in the non‐QIN wild populations, such as multidrug_multidrug_ABC_transporter, multidrug_mexT, multidrug_oprM, multidrug_mexF, multidrug_mexE, and multidrug_mexA, was higher when compared to other giant panda populations (Fig. 6). And the multidrug resistance genes were mainly identified in *Pseudomonas*, *Escherichia*, *Hafnia* and *Yersinia* (Fig. 6 and Fig. S2). We deduced that the divergence in the ARG in the gut microbiome in QIN population might be associated their long‐term isolation with non‐QIN populations.

Giant panda genomic research uncovered three significant genetic clusters (QIN, Minshan, and Qionglai–Daxiangling–Xiaoxiangling–Lianshang clusters), with the QIN and non‐QIN populations diverging approximately 0.3 million years ago (Zhao *et al*., 2013). Interestingly, the results of the host genome and gut microbiome and their ARGs revealed some consensus patterns. The pandas in the QIN Mountains consume more bamboo leaves (containing high levels of alkaloids) when compared to non‐QIN populations, which may result in some positive selection of various taste genes (Zhao *et al*., 2013). Alkaloids can increase butanol concentrations and shorten its fermentation period by some *Clostridium* strains (Shao and Chen, 2015). Moreover, clostrindolin, a product of some *Clostridium* strain, is an antimycobacterial pyrone alkaloid (Schieferdecker *et al*., 2019). These findings indicate that *Clostridium* species may utilize alkaloids. Here, the gut microbiome of the QIN population was found to have the highest proportion of *Clostridium* species (Table S4), as well as vancomycin resistance genes when compared to other wild and captive populations, which were mainly found in *Clostridium* species. Vancomycin is also widely used to treat infections with *Clostridium* strains (Zar *et al*., 2007). Strictly anaerobic *Clostridium* species have the potential ability to produce bioactive compounds, including potent antimicrobials (Pahalagedara *et al*., 2020). Therefore, we speculated that the long‐term isolation, selection pressure from the diet, and inner characteristics of *Clostridium* species might result in the geographic pattern observed in current ARGs identified in the gut microbiome of giant pandas, such as the high proportion of *Clostridium* and vancomycin resistance genes in the QIN population. Therefore, we provide an example of a potential consensus pattern between host population genetics and its symbiotic gut microbiome. We revealed that habitat isolation also impacts the ARG structure in the gut microbiome of mammals.

### Management and conservation

Our study revealed that captivity might lead to a special combination of ARGs as the treatment of captive individuals during normal health management affects the composition of ARGs. Thus, the difference in the ARG structure among different mammal groups in this study would provide some basic information for their management and conservation, especially for captive populations. The normal treatment of the mammals in the zoos (e.g. CA) should consider their potential tetracycline and macrolides resistance genes of the gut microbiome. Furthermore, maintaining the health of the captive giant panda population is important for the translocation of some small and isolated wild populations. The management of the captive giant panda population should think of the antibiotics resistance by *Escherichia*. Moreover, the care for the wild injured giant pandas should assess their mountain source and the potential antibiotic resistance by *Pseudomonas* (e.g. in non‐QIN mountain populations) and vancomycin resistance (e.g. in QIN mountain population).

## Experimental procedures

### Data collection

Considering the phylogeny position of the giant panda (belonging to Carnivora order), we collected the published metagenomes (raw data) of giant pandas and other carnivorans (Table S1). The 49 giant panda metagenomes came from five populations: three wild populations from the Qinling (Wu *et al*., 2017), Qionglai (Guo *et al*., 2019) and Xiaoxiangling Mountains (Zhu *et al*., 2018a), and two major captive populations from Yaan (Guo *et al*., 2019) and Chengdu (Zhang *et al*., 2018). In addition, we also integrated other published Carnivora metagenomes, including meat‐eating carnivorans (Zhu *et al*., 2018b), omnivorous carnivorans (Guo *et al*., 2018; Zhu *et al*., 2018b) and bamboo‐eating red pandas (Zhu *et al*., 2018a)), as well as 12 herbivore metagenomes (Zhu *et al*., 2018b) to investigate the effect of diet on the ARGs in the gut microbiome. The total number of species (subspecies) involved in this study was about 39 (Table S1). Most of the metagenome data used were from our previously published data, which can decrease the bias regarding the sequencing depth.

### Raw data treatment

Raw reads were filtered using Trimmomatic (Bolger *et al*., 2014) to remove (i) all read less than 50 bp in length, (ii) reads with degenerated bases (N's), and (iii) all duplicates defined as sequences whose initial 20 nucleotides were identical and shared an overall identity similarity of > 97% throughout the length of the shortest read. After blasting against the NR database in NCBI using diamond (Buchfink *et al*., 2014), we removed the putative host contamination. Megahit (Li *et al*., 2015) was used to assemble these clean reads into contigs. We used Prodigal (Hyatt *et al*., 2010) for gene prediction from the contigs and obtained the gene files per metagenome. Then, we used CD‐HIT (Li and Godzik, 2006) to construct non‐redundant gene sets with < 90% overlap and < 95% shared sequence identity from these gene files. Based on these gene profiles, we used salmon (Patro *et al*., 2015) to map the clean reads (keeping only the reads which theoretically belong to Prokaryotes) per metagenome to the clean non‐redundant gene profile and obtained the TPM (transcripts per million reads) abundance of these non‐redundant gene profiles in each metagenome. We also blasted these genes against the NR database in NCBI using diamond (Buchfink *et al*., 2014) and gained the putative taxon assignments of these genes per metagenome.

### The annotation of ARGs

We blasted the identified genes against the ARDB database using SARG2.0 with the default parameters (e‐value: 1e‐7; the cut‐off of blastx alignment identity: 60%; the blastx score: 60; and the cut‐off of blastx alignment length: 50%) (Yin *et al*., 2018) and gained the putative ARG assignment of these genes per metagenome. Then, we used custom Perl scripts to gain the abundance (TPM) of ARG types and subtypes for each metagenome.

### The relative abundance of gut microbiome communities and ARGs (type and subtype)

The abundance (TPM) of gut microbiome communities and ARGs per metagenome was transformed to relative abundance using STAMP (Parks *et al*., 2014). These relative abundance tables were then used for downstream analyses between groups, such abundance comparisons and cluster analysis.

### The bacteria taxon annotation of ARGs

The putative sequences of ARGs were blasted against the NR database in NCBI using diamond with these parameters (e‐value: 1e‐5; the cut‐off of blastx alignment identity: 55%; the blastx score: 60; and the cut‐off of blastx alignment length: 50%) (Buchfink *et al*., 2014), which led to the putative taxon assignments of these ARGs per metagenome. Then, we used custom Perl scripts to gain the abundance (TPM) of the taxon for these ARG types and subtype in each metagenome. The abundance (TPM) of gut microbiome communities and annotated ARGs per metagenome was transformed to relative abundance using STAMP (Parks *et al*., 2014).

### The contribution of taxon on these ARGs types and subtypes

Circos was used to visualized the contribution of bacteria taxon (at the genus level) regarding the ARG types and subtypes based on the relative abundance of bacteria genus for the annotated ARGs and the relative abundance of ARG types and subtypes in all annotated ARGs.

### The differences in the gut microbiome and ARGs between groups

The Jaccard distance for gut microbiome genus and ARGs (types and subtypes) relative abundance was used to generate non‐metric multidimensional scaling (NDMS) in PAST3 (Hammer *et al*., 2001). First, when using all 96 metagenomes, we separated them into four groups based on their diet (CA: meat‐eating carnivorans; OC, omnivorous carnivorans, Bamboo‐eating pandas (giant panda and red panda); HE, herbivores). Second, we only used the giant panda samples, which were divided into two groups based on the living environment (Wild and Captive panda populations). Lastly, we further evaluated the effect of geographic location in the giant panda samples, which were divided into five groups: QIN, wild Qinling population; QIO, wild Qionglai population; XXL, wild Xiaoxiangling population; CD, captive Chengdu population; and Yaan, captive Yaan population.

Moreover, to evaluate the effect of these factors on the composition of gut microbiota or ARG profiles, we performed one‐way PERMANOVA for Jaccard dissimilarities in species abundance using PAST3 (Hammer *et al*., 2001).

## Conflict of interest

The authors declare that they have no competing interests.

## Author contributions

LZ designed the research and wrote the manuscript, with input from QD, XG, XY and ZY. LZ, HC, TH, QD and ZZ conducted the metagenome data analysis. All authors read and approved the final manuscript.

## Ethical approval

Not applicable.

## Consent for publication

Not applicable.

## Supporting information


**Table S1.** Information regarding the samples used in this study.
**Table S2.** The genera summary of the nine groups formed from these 96 metagenomes.
**Table S3.** The mean abundance (%) of main ARG subtypes in the gut microbiome of giant pandas.
**Table S4.** The mean abundance (%) of the dominant putative *Clostridium* species in the gut microbiome of giant pandas.
**Fig. S1.** The distributions of dominant ARG subtypes and their abundances in the total annotated ARGs subtypes in the metagenomes.
**Fig. S2.** The distributions of ARG subtypes and their abundances in the total annotated ARGs subtypes in giant panda and red panda metagenomes.

## Data Availability

Not applicable.
