# Supplementary material for: Geographic pattern of antibiotic resistance genes in the metagenomes of the giant panda
Source: Microb Biotechnol. 2020 Aug 18;14(1):186–97. doi: 10.1111/1751-7915.13655 (PMC7888472; doi:10.1111/1751-7915.13655)
Supplement: Supplementary file 1 — Table S1. Information regarding the samples used in this study. Table S2. The genera summary of the nine groups formed from these 96 metagenomes. Table S3. The mean abundance (%) of main ARG subtypes in the gut microbiome of giant pandas. Table S4. The mean abundance (%) of the dominant putative Clostridium species in the gut microbiome of giant pandas. Fig. S1. The distributions of dominant ARG subtypes and their abundances in the total annotated ARGs subtypes in the metagenomes. Fig. S2. The distributions of ARG subtypes and their abundances in the total annotated ARGs subtypes in giant panda and red panda metagenomes. [file MBT2-14-186-s001.pdf]

Supporting information

Table S1 Information regarding the samples used in this study

SampleID	Diet	Living style*	Species*	Location*	Citation
SRR5415505	Bamboo-eating	Captive	Giant panda	Yaan	(Guo et al., 2019)
SRR5130540	Bamboo-eating	Captive	Giant panda	Yaan	(Guo et al., 2019)
SRR5415504	Bamboo-eating	Captive	Giant panda	Yaan	(Guo et al., 2019)
SRR5150002	Bamboo-eating	Captive	Giant panda	Yaan	(Guo et al., 2019)
SRR5149981	Bamboo-eating	Captive	Giant panda	Yaan	(Guo et al., 2019)
SRR5149936	Bamboo-eating	Captive	Giant panda	Yaan	(Guo et al., 2019)
SRR5149935	Bamboo-eating	Captive	Giant panda	Yaan	(Guo et al., 2019)
SRR5149886	Bamboo-eating	Captive	Giant panda	Yaan	(Guo et al., 2019)
SRR5130583	Bamboo-eating	Captive	Giant panda	Yaan	(Guo et al., 2019)
SRR5130565	Bamboo-eating	Captive	Giant panda	Yaan	(Guo et al., 2019)
ERX2429102	Bamboo-eating	Captive	Giant panda	Chengdu	(Zhang et al., 2018)
ERX2429101	Bamboo-eating	Captive	Giant panda	Chengdu	(Zhang et al., 2018)
ERX2429100	Bamboo-eating	Captive	Giant panda	Chengdu	(Zhang et al., 2018)
ERX2429099	Bamboo-eating	Captive	Giant panda	Chengdu	(Zhang et al., 2018)
ERX2429098	Bamboo-eating	Captive	Giant panda	Chengdu	(Zhang et al., 2018)
ERX2429087	Bamboo-eating	Captive	Giant panda	Chengdu	(Zhang et al., 2018)
ERX2333382	Bamboo-eating	Captive	Giant panda	Chengdu	(Zhang et al., 2018)
SAMC011136	Bamboo-eating	Wild	Giant panda	Qinling	(Wu et al., 2017)
SAMC011114	Bamboo-eating	Wild	Giant panda	Qinling	(Wu et al., 2017)
SAMC011133	Bamboo-eating	Wild	Giant panda	Qinling	(Wu et al., 2017)
SAMC011132	Bamboo-eating	Wild	Giant panda	Qinling	(Wu et al., 2017)
SAMC011131	Bamboo-eating	Wild	Giant panda	Qinling	(Wu et al., 2017)
SAMC011127	Bamboo-eating	Wild	Giant panda	Qinling	(Wu et al., 2017)
SAMC011121	Bamboo-eating	Wild	Giant panda	Qinling	(Wu et al., 2017)
SAMC011118	Bamboo-eating	Wild	Giant panda	Qinling	(Wu et al., 2017)
SAMC011115	Bamboo-eating	Wild	Giant panda	Qinling	(Wu et al., 2017)
SRR5149961	Bamboo-eating	Wild	Giant panda	Qionglai	(Guo et al., 2019)
SRR5149913	Bamboo-eating	Wild	Giant panda	Qionglai	(Guo et al., 2019)
SRR5149902	Bamboo-eating	Wild	Giant panda	Qionglai	(Guo et al., 2019)
SRR5149862	Bamboo-eating	Wild	Giant panda	Qionglai	(Guo et al., 2019)
SRR5130600	Bamboo-eating	Wild	Giant panda	Qionglai	(Guo et al., 2019)
SRR5130560	Bamboo-eating	Wild	Giant panda	Qionglai	(Guo et al., 2019)
SRR5130537	Bamboo-eating	Wild	Giant panda	Qionglai	(Guo et al., 2019)
20140128-zx-F03	Bamboo-eating	Wild	Giant panda	Xiaoxiangling	(Zhu et al., 2018b)
20131124-ZX-F03	Bamboo-eating	Wild	Giant panda	Xiaoxiangling	(Zhu et al., 2018b)
50	Bamboo-eating	Wild	Giant panda	Xiaoxiangling	(Zhu et al., 2018b)
34	Bamboo-eating	Wild	Giant panda	Xiaoxiangling	(Zhu et al., 2018b)
31	Bamboo-eating	Wild	Giant panda	Xiaoxiangling	(Zhu et al., 2018b)
9	Bamboo-eating	Wild	Giant panda	Xiaoxiangling	(Zhu et al., 2018b)
TT2	Bamboo-eating	Wild	Giant panda	Xiaoxiangling	(Zhu et al., 2018b)
19	Bamboo-eating	Wild	Giant panda	Xiaoxiangling	(Zhu et al., 2018b)
TT1	Bamboo-eating	Wild	Giant panda	Xiaoxiangling	(Zhu et al., 2018b)
LZP	Bamboo-eating	Wild	Giant panda	Xiaoxiangling	(Zhu et al., 2018b)
LX3	Bamboo-eating	Wild	Giant panda	Xiaoxiangling	(Zhu et al., 2018b)
CDXM40	Bamboo-eating	Wild	Giant panda	Xiaoxiangling	(Zhu et al., 2018b)
CDXM39	Bamboo-eating	Wild	Giant panda	Xiaoxiangling	(Zhu et al., 2018b)
CDXM27	Bamboo-eating	Wild	Giant panda	Xiaoxiangling	(Zhu et al., 2018b)
CDXM18	Bamboo-eating	Wild	Giant panda	Xiaoxiangling	(Zhu et al., 2018b)
23	Bamboo-eating	Wild	Giant panda	Xiaoxiangling	(Zhu et al., 2018b)
C2	Bamboo-eating	Wild	Red panda	Xiaoxiangling	(Zhu et al., 2018b)
C18	Bamboo-eating	Wild	Red panda	Xiaoxiangling	(Zhu et al., 2018b)
C14	Bamboo-eating	Wild	Red panda	Xiaoxiangling	(Zhu et al., 2018b)
B2-4	Bamboo-eating	Wild	Red panda	Xiaoxiangling	(Zhu et al., 2018b)
B1-2	Bamboo-eating	Wild	Red panda	Xiaoxiangling	(Zhu et al., 2018b)
A4	Bamboo-eating	Wild	Red panda	Xiaoxiangling	(Zhu et al., 2018b)
MJ22813045835_PY	HE	Captive	Argali	Beijing Zoo	(Zhu et al., 2018a)
MJ22813045835_BTCBY	HE	Captive	Hoolock.gibbon	Beijing Zoo	(Zhu et al., 2018a)
MJ22813045835_BJZML7	HE	Captive	Père David's deer	Beijing Zoo	(Zhu et al., 2018a)
LN	HE	Captive	Takin	Beijing Zoo	(Zhu et al., 2018a)

MJ22813045835_MHL	HE	Captive	Sika.deer	Beijing Zoo	(Zhu et al., 2018a)
MJ22813045835_MGYL	HE	Captive	Onager	Beijing Zoo	(Zhu et al., 2018a)
MJ22813045835_MAL	HE	Captive	Red.deer	Beijing Zoo	(Zhu et al., 2018a)
MJ22813045835_HYH	HE	Captive	Francois's Langur	Beijing Zoo	(Zhu et al., 2018a)
MJ22813045835_HJ	HE	Captive	Hairy.fronted.muntjac	Beijing Zoo	(Zhu et al., 2018a)
MJ22813045835_EHL	HE	Captive	Goitered.gazelle	Beijing Zoo	(Zhu et al., 2018a)
MJ22813045835_DJSH	HE	Captive	Black.snub.nosed.monkey	Beijing Zoo	(Zhu et al., 2018a)
MJ22813045835_BXCBY	HE	Captive	white.cheeked.gibbon	Beijing Zoo	(Zhu et al., 2018a)
NM	CA	Captive	Caracal	Beijing Zoo	(Zhu et al., 2018a)
MJ22813045835_CH	CA	Captive	Red.fox	Beijing Zoo	(Zhu et al., 2018a)
MJ22813045835_CA	CA	Captive	Dhole	Beijing Zoo	(Zhu et al., 2018a)
MJ22813045835_BHB-NJ	CA	Captive	Spotted.seal	Nanjing Underwater World Park	(Zhu et al., 2018a)
LB	CA	Captive	Cheetah	Beijing Zoo	(Zhu et al., 2018a)
HN13	CA	Captive	Indochinese.leopard	Beijing Zoo	(Zhu et al., 2018a)
HB	CA	Captive	Black.leopard	Beijing Zoo	(Zhu et al., 2018a)
FZS	CA	Captive	LionBJ	Beijing Zoo	(Zhu et al., 2018a)
DBH	CA	Captive	Siberian.tiger	Beijing Zoo	(Zhu et al., 2018a)
BXL	CA	Captive	Maned.wolf	Beijing Zoo	(Zhu et al., 2018a)
MJLH	CA	Captive	Bengal.tiger	Beijing Zoo	(Zhu et al., 2018a)
BLG	CA	Captive	Spotted.hyena	Beijing Zoo	(Zhu et al., 2018a)
MJ22813045835_YH	CA	Captive	Silver fox	Beijing Zoo	(Zhu et al., 2018a)
MJ22813045835_SH	CA	Captive	Corsac.fox	Beijing Zoo	(Zhu et al., 2018a)
MJ22813045835_NMHB-NJ	CA	Captive	Spotted.sealNJ2	Nanjing Underwater World Park	(Zhu et al., 2018a)
MJ22813045835_LH	CA	Captive	Arctic.fox	Beijing Zoo	(Zhu et al., 2018a)
MJ22813045835_HW2B	CA	Captive	Jaguar	Beijing Zoo	(Zhu et al., 2018a)
MJ22813045835_HBHL	CA	Captive	Black.backed.jackal	Beijing Zoo	(Zhu et al., 2018a)
MJ22813045835_GLG	CA	Captive	Striped hyena	Beijing Zoo	(Zhu et al., 2018a)
ZH	OC	Captive	Hog.badger	Beijing Zoo	(Zhu et al., 2018a)
MJ22813045835_MLX	OC	Captive	Sun.bear	Beijing Zoo	(Zhu et al., 2018a)
MJ22813045835_HX	OC	Captive	Raccoon	Beijing Zoo	(Zhu et al., 2018a)
SRR5130595	OC	Captive	Black bear	Yaan	(Guo et al., 2018)
SRR5130563	OC	Captive	Black bear	Yaan	(Guo et al., 2018)
SRR5130533	OC	Captive	Black bear	Yaan	(Guo et al., 2018)
SRR5130531	OC	Captive	Black bear	Yaan	(Guo et al., 2018)
SRR5130527	OC	Captive	Black bear	Yaan	(Guo et al., 2018)
MJ22813045835_ZX	OC	Captive	Brow.bear	Beijing Zoo	(Zhu et al., 2018a)
MJ22813045835_MX	OC	Captive	Kinkajou	Beijing Zoo	(Zhu et al., 2018a)

*The group analysis only included giant panda samples.

CA, meat-eating carnivorans. OC, omnivorous carnivorans. HE, herbivores. Chengdu, Chengdu giant panda Breeding center. Yaan, giant panda research center in Yaan.

Table S2 The genera summary of the nine groups formed from these 96 metagenomes

Genus	CA (%)	OC (%)	Qinling (%)	Qionglai (%)	XXL (%)	Yaan(%)	Chengdu (%)	RPxxl (%)	HE (%)
p__Proteobacteria;c__Gammaproteobacteria;o__Pseudomonadales;f__Pseudomonadaceae;g__Pseudomonas	0.07358	4.32976	0.01399	22.06394	43.70908	0.29144	12.32207	61.37176	0.04291
p__Proteobacteria;c__Gammaproteobacteria;o__Enterobacterales;f__Enterobacteriaceae;g__Escherichia	2.69980	17.62788	10.08703	6.53788	5.78505	33.54275	28.12609	0.26900	0.10564
p__Firmicutes;c__Clostridia;o__Clostridiales;f__Clostridiaceae;g__Clostridium	4.04778	8.27262	41.62053	0.15433	16.80261	3.88416	0.11267	0.21159	6.49282
p__Firmicutes;c__Bacilli;o__Lactobacillales;f__Streptococcaceae;g__Streptococcus	0.21572	15.89632	12.32702	0.31717	6.99485	26.81897	0.94633	8.84151	0.18720
p__Bacteroidetes;c__Bacteroidia;o__Bacteroidales;f__Bacteroidaceae;g__Bacteroides	30.05432	0.04400	0.03108	0.03909	0.01020	0.31254	0.01657	0.00390	10.26886
p__Proteobacteria;c__Gammaproteobacteria;o__Enterobacterales;f__Enterobacteriaceae;Unclassified	0.61820	4.36630	2.14696	1.12846	1.07172	6.55898	9.86128	0.03478	0.00170
p__Firmicutes;c__Bacilli;o__Lactobacillales;f__Lactobacillaceae;g__Lactobacillus	0.16674	4.68659	2.30370	0.02553	0.13552	3.26846	13.50225	0.05892	0.12182
p__Proteobacteria;Unclassified;Unclassified;Unclassified;Unclassified	0.76935	4.12680	1.22728	0.79798	0.98647	7.11008	4.04970	0.08788	0.00116
p__Proteobacteria;c__Gammaproteobacteria;o__Enterobacterales;f__Enterobacteriaceae;g__Klebsiella	0.27852	0.89783	0.34717	0.34818	0.24747	1.03669	13.28608	0.04107	0.07713
p__Bacteroidetes;c__Bacteroidia;o__Bacteroidales;f__Prevotellaceae;g__Prevotella	5.43477	0.00536	0.00836	0.01047	0.00499	0.00146	0.00016	0.00069	10.90080
p__Proteobacteria;c__Gammaproteobacteria;o__Enterobacterales;f__Yersiniaceae;g__Yersinia	0.00140	0.14457	2.70325	6.26697	5.08232	0.55837	0.03375	0.37640	0.00114
p__Firmicutes;c__norank;o__norank;f__norank;g__norank	1.88147	0.02723	0.06363	0.04409	0.00808	0.01148	0.00445	0.00081	9.63561
p__Proteobacteria;c__Betaproteobacteria;o__Burkholderiales;f__Oxalobacteraceae;g__Janthinobacterium	0.00623	0.27698	0.00000	1.57858	0.68921	0.06270	0.00018	8.82555	0.00034
p__Firmicutes;c__Erysipelotrichia;o__Erysipelotrichales;f__Erysipelotrichaceae;g__Turicibacter	0.01117	0.37365	9.03083	0.00436	0.43724	0.20080	0.00001	0.00880	0.01634
p__Firmicutes;c__Clostridia;o__Clostridiales;f__Lachnospiraceae;g__Blautia	7.49702	0.28244	0.06475	0.00415	0.09126	0.02766	0.04678	0.00218	0.96182
p__Proteobacteria;c__Gammaproteobacteria;o__Enterobacterales;f__Hafniaceae;g__Hafnia	0.00086	0.04227	0.29978	6.53067	1.84538	0.02450	0.00371	0.02067	0.00046
p__Firmicutes;c__Bacilli;o__Lactobacillales;f__Enterococcaceae;g__Enterococcus	0.17529	1.20347	0.37748	0.00687	0.17831	0.57880	0.62138	5.37606	0.09168
p__Bacteroidetes;c__Flavobacteriia;o__Flavobacteriales;f__Flavobacteriaceae;g__Flavobacterium	0.00446	0.23696	0.00022	6.63691	1.02123	0.05485	0.00005	0.59172	0.04916
p__Fusobacteria;c__Fusobacteriia;o__Fusobacteriales;f__Fusobacteriaceae;g__Fusobacterium	7.21399	0.42385	0.01395	0.00188	0.00441	0.00486	0.00646	0.00126	0.14064
p__Proteobacteria;c__Gammaproteobacteria;o__Enterobacterales;f__Enterobacteriaceae;g__Shigella	0.20161	1.15527	0.44747	0.52982	0.29430	2.42339	2.18226	0.04787	0.08648
p__Firmicutes;c__Bacilli;o__Lactobacillales;f__Streptococcaceae;g__Lactococcus	0.01076	0.15741	6.76255	0.00042	0.01228	0.05028	0.06204	0.12692	0.02992
p__Proteobacteria;c__Gammaproteobacteria;o__Enterobacterales;f__Enterobacteriaceae;g__Citrobacter	0.07084	3.46651	0.48986	0.21308	0.13315	0.58277	1.15356	0.00650	0.00366
p__Bacteroidetes;c__Sphingobacteriia;o__Sphingobacteriales;f__Sphingobacteriaceae;g__Pedobacter	0.00187	0.07399	0.00001	0.97192	2.74604	0.21521	0.00003	1.91878	0.01851
p__Bacteroidetes;c__Bacteroidia;o__Bacteroidales;f__Rikenellaceae;g__Alistipes	0.45549	0.00163	0.00012	0.00967	0.00011	0.00007	0.00009	0.00005	5.38200
Unclassified;Unclassified;Unclassified;Unclassified;Unclassified	0.87116	0.60996	0.36452	0.17680	0.25793	1.21143	1.20343	0.09052	0.35486
p__Proteobacteria;c__Alphaproteobacteria;o__Rhizobiales;f__Bradyrhizobiaceae;g__Bradyrhizobium	0.00026	0.35056	0.00000	4.73171	0.00445	0.00335	0.00007	0.00575	0.00245

p__Proteobacteria;c__Betaproteobacteria;o__Burkholderiales;f__Oxalobacteraceae;g__Duganella	0.00013	1.94987	0.00000	0.97843	0.32934	0.03454	0.00006	1.43057	0.00056
p__norank;c__norank;o__norank;f__norank;g__norank	1.29876	0.18030	0.03110	0.25601	0.05565	0.17618	0.07021	0.05378	2.52936
p__Proteobacteria;c__Gammaproteobacteria;o__Enterobacterales;Unclassified;Unclassified	0.01547	0.24204	0.11726	0.17571	0.08317	0.29736	3.59752	0.00549	0.00011
p__Fusobacteria;c__Fusobacteriia;o__Fusobacteriales;f__Fusobacteriaceae;g__Cetobacterium	0.73820	3.63055	0.00022	0.00002	0.00003	0.02099	0.00000	0.00000	0.00216
p__Firmicutes;c__Clostridia;o__Clostridiales;f__norank;g__norank	0.49464	0.03642	0.06391	0.02948	0.12797	0.05631	0.00372	0.01141	3.53764
p__Proteobacteria;c__Betaproteobacteria;o__Burkholderiales;f__Sutterellaceae;g__Sutterella	4.16008	0.00398	0.00001	0.00896	0.00203	0.00122	0.00000	0.02095	0.05238
p__Firmicutes;c__Clostridia;o__Clostridiales;f__Ruminococcaceae;g__Ruminococcus	0.86877	0.01844	0.07085	0.00924	0.05372	0.21776	0.01976	0.01350	2.80809
p__Proteobacteria;c__Gammaproteobacteria;o__Enterobacterales;f__Enterobacteriaceae;g__Salmonella	0.10744	0.96946	0.29269	0.51718	0.17102	1.19081	0.75258	0.00538	0.00540
p__Proteobacteria;c__Gammaproteobacteria;o__Xanthomonadales;f__Xanthomonadaceae;g__Stenotrophomonas	0.00198	0.03510	0.00000	3.40728	0.11677	0.01450	0.15582	0.23724	0.00067
p__Actinobacteria;c__Actinobacteria;o__Micrococcales;f__Microbacteriaceae;g__Cryobacterium	0.00002	0.00045	0.00001	0.06219	0.15819	0.00215	0.00001	3.51042	0.00035
p__Proteobacteria;c__Gammaproteobacteria;o__norank;f__norank;g__norank	0.00040	2.60731	0.00076	0.61142	0.03426	0.00164	0.00882	0.04132	0.01482
p__Firmicutes;c__Bacilli;o__Bacillales;f__Bacillaceae;g__Bacillus	0.50071	0.30906	0.90331	0.05963	0.39777	0.12793	0.22067	0.04188	0.57319
p__Proteobacteria;c__Betaproteobacteria;o__Burkholderiales;f__Burkholderiaceae;g__Paraburkholderia	0.00533	2.83195	0.00062	0.23048	0.02335	0.00310	0.00058	0.02593	0.00112
p__Proteobacteria;c__Gammaproteobacteria;o__Enterobacterales;f__Enterobacteriaceae;g__Enterobacter	0.03225	0.56077	0.16128	0.83268	0.21007	0.56431	0.60682	0.01033	0.00781
p__Actinobacteria;c__Coriobacteriia;o__Coriobacteriales;f__Coriobacteriaceae;g__Collinsella	2.39884	0.00392	0.00011	0.00006	0.00050	0.00000	0.48505	0.00000	0.08219
p__Firmicutes;c__Clostridia;o__Clostridiales;f__Eubacteriaceae;g__Eubacterium	0.59875	0.03879	0.02446	0.00201	0.00397	0.03074	0.00311	0.00476	2.02363
p__Bacteroidetes;c__Bacteroidia;o__Bacteroidales;f__norank;g__norank	0.17144	0.00110	0.00012	0.00357	0.00025	0.00018	0.00004	0.00008	2.44687
p__Firmicutes;c__Clostridia;o__Clostridiales;f__Lachnospiraceae;g__norank	0.92214	0.08113	0.09874	0.00315	0.06140	0.03156	0.00290	0.00410	1.40136
p__Proteobacteria;c__Gammaproteobacteria;o__Enterobacterales;f__norank;g__Plesiomonas	0.01270	2.39314	0.00000	0.00942	0.00068	0.09814	0.08385	0.00008	0.00024
p__Proteobacteria;c__Gammaproteobacteria;o__Enterobacterales;f__Hafniaceae;Unclassified	0.00001	0.00334	0.10336	2.00611	0.43139	0.00641	0.00034	0.00401	0.00000
p__Firmicutes;c__Clostridia;o__Clostridiales;f__Ruminococcaceae;g__norank	0.16258	0.00716	0.01378	0.00827	0.03151	0.00200	0.01289	0.02123	2.17179
p__Bacteroidetes;c__Bacteroidia;o__Bacteroidales;Unclassified;Unclassified	1.78157	0.00100	0.00000	0.00135	0.00016	0.00656	0.00051	0.00005	0.61905
p__Proteobacteria;c__Gammaproteobacteria;o__Enterobacterales;f__Yersiniaceae;g__Rahnella	0.00045	0.84617	0.19703	0.51020	0.43550	0.06944	0.00289	0.23719	0.00003
p__Firmicutes;c__Bacilli;o__Bacillales;f__Staphylococcaceae;g__Staphylococcus	0.81787	0.42949	0.03433	0.00220	0.06748	0.07058	0.23725	0.03397	0.56255
p__Proteobacteria;c__Gammaproteobacteria;o__Enterobacterales;f__Yersiniaceae;g__Serratia	0.00966	0.18768	0.16166	1.25080	0.25028	0.24670	0.07909	0.03970	0.00073
p__Proteobacteria;c__Betaproteobacteria;o__Burkholderiales;f__Comamonadaceae;g__Comamonas	0.03298	0.03159	0.00002	1.86596	0.05067	0.01547	0.18139	0.04523	0.00203
p__Bacteroidetes;c__Bacteroidia;o__Bacteroidales;f__Tannerellaceae;g__Parabacteroides	1.02646	0.00860	0.00078	0.00561	0.00369	0.00447	0.00041	0.00114	1.09934
p__Proteobacteria;c__Betaproteobacteria;o__Burkholderiales;f__Alcaligenaceae;g__Achromobacter	0.00966	0.02685	0.00000	1.98370	0.02706	0.00056	0.00121	0.00787	0.00612
p__Proteobacteria;c__Epsilonproteobacteria;o__Campylobacterales;f__Campylobacteraceae;g__Campylobacter	0.60123	0.00970	0.05813	0.00777	0.05728	0.70218	0.46688	0.00280	0.05691

p__Firmicutes;c__Clostridia;o__Clostridiales;Unclassified;Unclassified	0.70187	0.04687	0.12773	0.00397	0.16039	0.08089	0.02149	0.00278	0.73493
p__Firmicutes;c__Bacilli;o__Bacillales;f__Bacillaceae;g__Lysinibacillus	0.00338	0.00547	0.17259	0.42094	1.18935	0.00863	0.00001	0.01938	0.05961
p__Firmicutes;c__Clostridia;o__Clostridiales;f__Oscillospiraceae;g__Oscillibacter	0.38321	0.00385	0.00042	0.01964	0.00010	0.00015	0.00019	0.00002	1.47092
p__Firmicutes;c__Negativicutes;o__Selenomonadales;f__Selenomonadaceae;g__Megamonas	1.80513	0.00969	0.04329	0.00091	0.00177	0.00115	0.00024	0.00008	0.01477
p__Proteobacteria;c__Alphaproteobacteria;o__Sphingomonadales;f__Sphingomonadaceae;g__Novosphingobium	0.00170	1.22727	0.00000	0.55795	0.00475	0.00165	0.00000	0.00181	0.00609
p__Firmicutes;c__Negativicutes;o__Veillonellales;f__Veillonellaceae;g__Megasphaera	0.02356	0.00146	0.00019	0.00065	0.02024	0.00248	1.60335	0.00001	0.08902
p__Firmicutes;c__Clostridia;o__Clostridiales;f__Ruminococcaceae;g__Faecalibacterium	0.30162	0.02311	0.01198	0.01735	0.01466	0.02139	0.00108	0.00001	1.32803
p__Firmicutes;c__Negativicutes;o__Acidaminococcales;f__Acidaminococcaceae;g__Phascolarctobacterium	1.00398	0.00089	0.00041	0.00155	0.00001	0.00006	0.00016	0.00020	0.70349
p__Proteobacteria;c__Gammaproteobacteria;o__Enterobacterales;f__Enterobacteriaceae;g__Lelliottia	0.00210	0.04918	0.00511	1.36528	0.17802	0.02687	0.01727	0.00165	0.00003
p__Firmicutes;c__Clostridia;o__Clostridiales;f__Lachnospiraceae;g__Lachnoclostridium	0.68971	0.02187	0.08243	0.00250	0.04209	0.02565	0.01856	0.00014	0.72506
p__Proteobacteria;c__Gammaproteobacteria;o__Pasteurellales;f__Pasteurellaceae;g__Actinobacillus	0.00278	0.00125	0.10291	0.00091	0.00454	1.48070	0.00042	0.00095	0.00196
p__Proteobacteria;c__Deltaproteobacteria;o__Myxococcales;f__norank;g__norank	0.00001	0.01121	0.00000	1.55528	0.00008	0.00048	0.00000	0.00013	0.00074
p__Fibrobacteres;c__Fibrobacteria;o__Fibrobacterales;f__Fibrobacteraceae;g__Fibrobacter	0.03118	0.00254	0.00017	0.00292	0.00026	0.00001	0.00000	0.00000	1.39749
p__Firmicutes;c__Clostridia;o__Clostridiales;f__Lachnospiraceae;g__Roseburia	0.38363	0.00623	0.01354	0.00119	0.00237	0.01238	0.00091	0.00032	0.99113
p__Bacteroidetes;c__Sphingobacteriia;o__Sphingobacteriales;f__Sphingobacteriaceae;g__Sphingobacterium	0.00114	0.00121	0.00005	0.64173	0.62943	0.03351	0.00454	0.02977	0.01635
p__Proteobacteria;c__Betaproteobacteria;o__Burkholderiales;f__Comamonadaceae;g__Acidovorax	0.00224	0.05552	0.00004	1.15303	0.05700	0.00914	0.00667	0.06052	0.00135
p__Firmicutes;c__Clostridia;o__Clostridiales;f__Lachnospiraceae;g__Coprococcus	0.84063	0.02212	0.01981	0.00221	0.05445	0.01759	0.00371	0.02667	0.35502
p__Proteobacteria;c__Gammaproteobacteria;o__Pseudomonadales;f__Moraxellaceae;g__Psychrobacter	0.01609	1.27250	0.00028	0.00047	0.00220	0.00991	0.03249	0.00057	0.00242
p__Actinobacteria;c__Actinobacteria;o__Bifidobacteriales;f__Bifidobacteriaceae;g__Bifidobacterium	0.04613	0.97585	0.00468	0.01861	0.00180	0.01363	0.21769	0.00045	0.05568
p__Chlamydiae;c__Chlamydiia;o__Chlamydiales;f__Chlamydiaceae;g__Chlamydia	0.29085	0.08670	0.03876	0.00630	0.02617	0.01056	0.00450	0.73270	0.13347
p__Proteobacteria;c__Betaproteobacteria;o__Burkholderiales;f__Comamonadaceae;g__Variovorax	0.00068	0.09321	0.00000	0.85223	0.07143	0.00547	0.00172	0.27018	0.00117
p__Spirochaetes;c__Spirochaetia;o__Spirochaetales;f__Spirochaetaceae;g__Treponema	0.00906	0.00258	0.00435	0.00354	0.00248	0.00067	0.00010	0.00065	1.23085
p__Proteobacteria;c__Gammaproteobacteria;o__Enterobacterales;f__Morganellaceae;g__Morganella	0.00070	0.00429	0.00639	0.02038	0.00815	1.03209	0.11064	0.00059	0.00010
p__Firmicutes;c__Clostridia;o__Clostridiales;f__Peptostreptococcaceae;g__Clostridioides	0.27861	0.22666	0.30699	0.00637	0.16703	0.05724	0.00216	0.00433	0.12835
p__Proteobacteria;c__Gammaproteobacteria;o__Pseudomonadales;f__Moraxellaceae;g__Acinetobacter	0.09626	0.09890	0.08169	0.44399	0.20899	0.10928	0.00623	0.02552	0.06385
p__Bacteroidetes;c__norank;o__norank;f__norank;g__norank	0.03332	0.00092	0.00009	0.02986	0.00953	0.00046	0.00015	0.00294	0.96946
p__Proteobacteria;c__Betaproteobacteria;o__Neisseriales;f__Neisseriaceae;g__Neisseria	0.62469	0.00334	0.01756	0.00602	0.01675	0.11573	0.21465	0.00706	0.00420
p__Proteobacteria;c__Epsilonproteobacteria;o__Campylobacterales;f__Helicobacteraceae;g__Helicobacter	0.17503	0.29319	0.15869	0.00179	0.10141	0.05577	0.07747	0.00081	0.14330
p__Firmicutes;c__Bacilli;o__Lactobacillales;f__Carnobacteriaceae;g__Carnobacterium	0.00159	0.01247	0.02900	0.00358	0.04189	0.00444	0.00041	0.90706	0.00337

p__Actinobacteria;c__Actinobacteria;o__Micrococcales;f__Micrococcaceae;g__Arthrobacter	0.00064	0.00181	0.00009	0.19433	0.17167	0.01637	0.00035	0.61059	0.00179
p__Proteobacteria;c__Alphaproteobacteria;o__Sphingomonadales;f__Sphingomonadaceae;g__Sphingomonas	0.00041	0.14195	0.00019	0.71284	0.02883	0.00267	0.00001	0.08552	0.00236
p__Firmicutes;c__Clostridia;o__Clostridiales;f__Peptostreptococcaceae;g__Romboutsia	0.05986	0.55338	0.25489	0.00151	0.05172	0.01882	0.00000	0.00171	0.02007
p__Bacteroidetes;c__Bacteroidia;o__Bacteroidales;f__Muribaculaceae;g__norank	0.52253	0.00072	0.00000	0.00121	0.00002	0.00000	0.00001	0.00003	0.43372
p__Firmicutes;c__Clostridia;o__norank;f__norank;g__norank	0.12771	0.00296	0.01045	0.00818	0.00144	0.00297	0.00009	0.00014	0.79771
p__Firmicutes;c__Clostridia;o__Clostridiales;f__Ruminococcaceae;g__Flavonifractor	0.29279	0.00902	0.00590	0.00554	0.00230	0.01726	0.00165	0.00009	0.60492
p__Firmicutes;c__Clostridia;o__Clostridiales;f__Ruminococcaceae;g__Anaerotruncus	0.08137	0.01269	0.00875	0.00327	0.08108	0.01426	0.00068	0.00001	0.70487
p__Proteobacteria;c__Gammaproteobacteria;Unclassified;Unclassified;Unclassified	0.02812	0.28952	0.04872	0.01552	0.01506	0.17879	0.31464	0.00126	0.00051
p__Firmicutes;c__Bacilli;o__Lactobacillales;f__Leuconostocaceae;g__Leuconostoc	0.02797	0.44961	0.02882	0.05931	0.11834	0.00564	0.17167	0.00343	0.00213
p__Proteobacteria;c__Gammaproteobacteria;o__Enterobacterales;f__Morganellaceae;g__Providencia	0.01441	0.14147	0.23624	0.28318	0.00990	0.09026	0.02697	0.00151	0.00846
p__Proteobacteria;c__Betaproteobacteria;o__Burkholderiales;f__Oxalobacteraceae;g__Herbaspirillum	0.00017	0.56873	0.00001	0.17764	0.00752	0.00066	0.00022	0.04216	0.00102
p__Firmicutes;c__Clostridia;o__Clostridiales;f__Lachnospiraceae;Unclassified	0.53268	0.00875	0.00602	0.00044	0.02737	0.00066	0.00172	0.00349	0.20403
p__Firmicutes;Unclassified;Unclassified;Unclassified;Unclassified	0.28012	0.06673	0.09284	0.00142	0.01514	0.05424	0.01003	0.00186	0.25211
p__Proteobacteria;c__norank;o__norank;f__norank;g__norank	0.17760	0.03033	0.00007	0.48578	0.00825	0.00048	0.00299	0.00448	0.05908
p__Firmicutes;c__Clostridia;o__Clostridiales;f__Lachnospiraceae;g__Dorea	0.35185	0.01975	0.01115	0.00288	0.02405	0.03943	0.01108	0.00263	0.29430
p__Proteobacteria;c__Gammaproteobacteria;o__Enterobacterales;f__Enterobacteriaceae;g__Enterobacillus	0.00179	0.02082	0.02100	0.58521	0.10965	0.00301	0.00016	0.00094	0.00051
p__Firmicutes;c__Clostridia;o__Clostridiales;f__Ruminococcaceae;g__Pseudoflavonifractor	0.11681	0.00332	0.00112	0.00811	0.00032	0.17446	0.00018	0.00006	0.42012
p__Proteobacteria;c__Betaproteobacteria;o__Burkholderiales;f__Oxalobacteraceae;g__Massilia	0.00025	0.09077	0.00000	0.11904	0.19125	0.00421	0.00007	0.31617	0.00257
p__Proteobacteria;c__Betaproteobacteria;o__Burkholderiales;f__Comamonadaceae;g__Polaromonas	0.00017	0.02085	0.00006	0.67542	0.00514	0.00093	0.00094	0.00898	0.00043
p__Firmicutes;c__Clostridia;o__Clostridiales;f__Lachnospiraceae;g__Cellulosilyticum	0.00454	0.00639	0.61400	0.00072	0.04084	0.01462	0.00004	0.00049	0.02656
p__Bacteroidetes;c__Chitinophagia;o__Chitinophagales;f__Chitinophagaceae;g__Chitinophaga	0.04510	0.52541	0.00016	0.09107	0.00965	0.00055	0.00001	0.01961	0.01294
p__Firmicutes;c__Bacilli;o__Bacillales;f__Paenibacillaceae;g__Paenibacillus	0.03087	0.03023	0.15491	0.10538	0.06513	0.09693	0.04166	0.03282	0.14504
p__Bacteroidetes;c__Bacteroidia;o__Bacteroidales;f__Prevotellaceae;g__Prevotellamassilia	0.51951	0.00023	0.00000	0.00078	0.00000	0.00000	0.00000	0.00000	0.17796
p__Proteobacteria;c__Deltaproteobacteria;o__Myxococcales;f__norank;g__Minicystis	0.00000	0.01165	0.00000	0.67213	0.00007	0.00007	0.00000	0.00012	0.00015
p__Firmicutes;c__Clostridia;o__Clostridiales;f__Lachnospiraceae;g__Butyrivibrio	0.03838	0.01945	0.01788	0.00041	0.00158	0.01368	0.00075	0.00001	0.58289
p__Firmicutes;c__Clostridia;o__Clostridiales;f__Ruminococcaceae;g__Subdoligranulum	0.06611	0.01674	0.00057	0.00056	0.06246	0.05218	0.00029	0.00004	0.45823
Others	9.39419	5.87079	4.19087	14.67722	4.19506	3.22902	2.53110	2.63317	19.17168

Others, including the low mean abundance genera and unclassified groups.

CA, meat-eating carnivorans. OC, omnivorous carnivorans. HE, herbivores. Chengdu, Chengdu giant panda Breeding center. Yaan, giant panda research center in Yaan. XXL, wild Xiaoxiangliang giant panda population.

RPxxl, wild Xiaoxiangling Red panda population.

Table S3 The mean abundance (%) of main ARG subtypes in the gut microbiome of giant pandas

	Wild	Wild	Wild	Captive	Captive
**Subtype**	**Qinling**	**Qionglai**	**XXL**	**YAAN**	**Chengdu**
bacitracin__bacA	21.51	4.29	7.36	7.33	2.91
multidrug__multidrug_transporter	5.29	7.21	6.00	6.97	2.40
vancomycin__vanS	21.39	0.47	4.66	0.08	0.05
multidrug__ompF	2.94	2.10	1.11	6.11	3.71
unclassified__cAMP-regulatory protein	1.66	2.55	3.89	3.21	2.79
multidrug__omp36	2.14	3.14	1.19	4.24	3.27
fosmidomycin__rosA	0.92	4.79	4.64	1.23	2.31
multidrug__acrB	1.63	3.81	2.39	1.98	3.47
tetracycline__tet34	2.01	2.27	0.96	4.41	2.10
multidrug__multidrug_ABC_transporter	0.88	2.01	8.56	0.12	0.05
unclassified__DNA-binding_protein_H-NS	2.03	2.65	0.89	2.44	2.39
multidrug__mdtB	0.56	4.63	2.95	0.73	1.39
multidrug__emrB	1.16	2.31	0.99	2.09	1.74
unclassified__transcriptional regulatory protein CpxR cpxR	1.16	1.36	0.60	2.75	2.39
multidrug__mdtC	0.48	4.45	1.34	0.75	0.94
beta-lactam__PBP-1A	2.41	0.03	1.00	4.36	0.02
multidrug__ompR	0.67	1.95	1.47	1.16	1.93
polymyxin__arnA	0.48	1.48	2.66	0.82	1.68
multidrug__acrA	0.99	1.22	0.70	1.74	2.37
multidrug__mexF	0.03	3.14	2.53	0.06	1.01
multidrug__emrD	0.91	1.27	0.84	2.19	1.52
multidrug__emrE	1.20	1.22	0.83	1.01	2.33
multidrug__mexT	0.00	1.40	4.20	0.02	0.89
multidrug__mexX	0.63	0.86	1.62	1.45	1.73
fosmidomycin__rosB	0.78	1.77	0.79	1.43	1.32
unclassified__truncated putative response regulator ArlR	2.87	0.06	1.01	0.77	1.07
unclassified__bacterial regulatory protein LuxR	1.04	0.79	0.34	2.08	1.26
unclassified__cob(I)alamin adenolsyltransferase	0.90	1.44	0.60	1.32	1.21
multidrug__mdtG	0.66	1.13	0.32	1.46	1.84
multidrug__TolC	0.83	1.15	0.71	1.38	1.32
multidrug__oprM	0.03	1.20	3.46	0.02	0.68
multidrug__EmrB-QacA family major facilitator transporter	0.24	0.37	0.12	3.47	0.98
multidrug__mdfA	0.77	1.39	0.74	1.02	1.21
kasugamycin__kasugamycin resistance protein ksgA	0.77	0.99	0.38	1.77	1.18
macrolide-lincosamide-streptogramin__macB	0.56	1.46	0.86	1.11	1.07
multidrug__mdtH	0.68	1.07	0.44	1.23	1.52
multidrug__mexE	0.05	1.35	2.52	0.05	0.76
multidrug__mdtE	0.55	0.82	0.34	1.61	1.31
multidrug__emrA	0.75	1.10	0.49	1.05	1.12

multidrug__mdtK	0.55	1.08	0.64	1.16	1.02
multidrug__bicyclomycin-multidrug_efflux_protein_bcr	0.61	1.01	0.48	1.06	1.18
unclassified__DNA-binding transcriptional regulator gadX	1.09	0.34	0.34	1.95	0.60
macrolide-lincosamide-streptogramin__macA	0.62	0.79	0.43	0.98	1.35
multidrug__mdtL	0.72	0.30	0.29	1.16	1.68
multidrug__mdtM	0.45	0.09	0.20	1.70	1.66
multidrug__mexA	0.00	0.88	2.27	0.00	0.83
multidrug__mdtA	0.64	1.05	0.50	0.82	0.82
macrolide-lincosamide-streptogramin__ermB	0.00	0.00	0.00	0.03	3.75
beta-lactam__class C beta-lactamase	0.49	0.44	0.96	1.14	0.62
tetracycline__tetD	0.87	0.45	0.46	0.86	0.98

Table S4 The mean abundance (%) of the dominant putative Clostridium species in the gut microbiome of giant pandas

Specie	Wild Qinling	Wild Qionglai	Wild XXL	Captive Chengdu	Captive Yaan
Clostridium cuniculi	9.7014	0.0434	6.2472	0.0024	0.9893
Clostridium perfringens	9.6861	0.0039	0.1806	0.0102	0.8036
Clostridium_unclassified	3.7258	0.0112	1.7093	0.0052	0.1656
Clostridium nigeriense	3.2707	0.0042	1.6635	0.0000	0.0755
Clostridium ventriculi	0.1539	0.0047	0.0210	0.0002	0.2923
Clostridiales bacterium	0.0087	0.0122	0.0953	0.0007	0.0186
Clostridium botulinum	0.8725	0.0095	0.6295	0.0025	0.3902
Clostridium disporicum	1.0338	0.0040	0.6611	0.0002	0.1117
Clostridiales_unclassified	0.1277	0.0040	0.1604	0.0215	0.0809
Clostridium beijerinckii	1.2934	0.0045	0.4179	0.0005	0.0323
Clostridium celatum	0.8371	0.0033	0.5128	0.0002	0.0865
Clostridium saccharobutylicum	1.1347	0.0030	0.3337	0.0000	0.0083
Clostridium tertium	0.8453	0.0026	0.3699	0.0000	0.0271
Clostridium sp. CAG:413	0.0000	0.0005	0.0000	0.0001	0.0001
Clostridioides difficile	0.2961	0.0063	0.1660	0.0022	0.0562
Clostridium sp. CAG:465	0.0000	0.0000	0.0000	0.0000	0.0000
Clostridium sp. CAG:1219	0.0000	0.0000	0.0000	0.0000	0.0000
Clostridium sp. AF17-2	0.0000	0.0000	0.0000	0.0000	0.0000
Clostridium sp. AF34-13	0.0005	0.0000	0.0001	0.0000	0.0006
Clostridium isatidis	0.7773	0.0017	0.0692	0.0000	0.0048

Figure S1 The distributions of dominant ARG subtypes and their abundances in the total annotated ARGs subtypes in the metagenome (visualized by Circos(Krzywinski et al., 2009)). The length of the bars on the outer-ring represents the percentage of gut microbiome groups (genera level) in each ARGs subtype. Each gut microbiome genus was represented by a specific ribbon color, and the width of each ribbon demonstrates the abundance of each genus in the ARGs subtype. **A.** CA (meat-eating carnivorans). **B.** OC (omnivorous carnivorans). **C,** HE (herbivores). amin, aminoglycoside. bac, bacitracin. beta, beta-lactam. ble, bleomycin. chl, chloramphenicol. fosm, fosmidomycin. kas, kasugamycin. mac, macrolide-lincosamide-streptogramin. mul, multidrug. poly, polymyxin. sul, sulfonamide. tet, tetracycline. tri, trimethoprim. unc, unclassified. van, vancomycin.

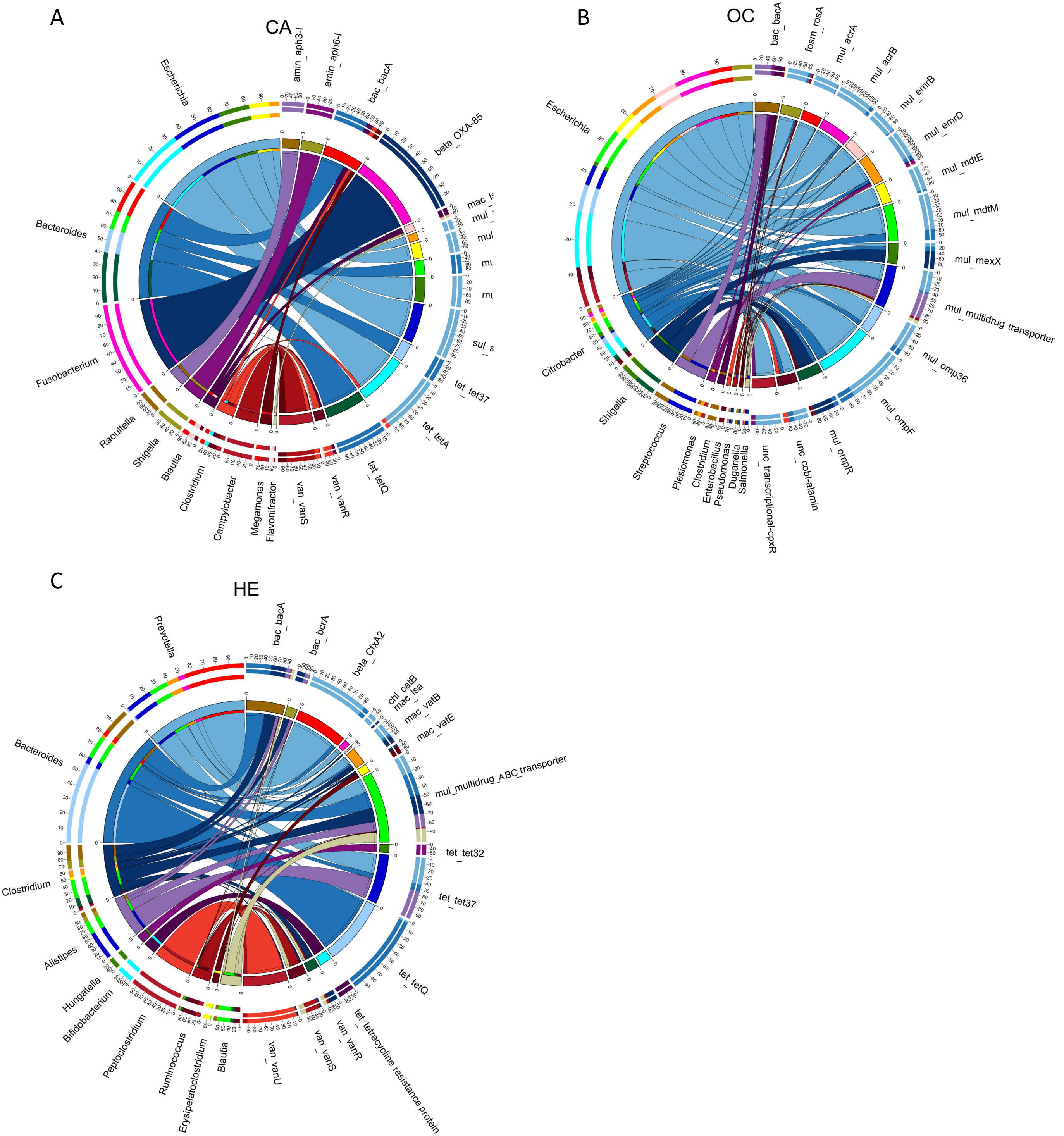
A
CA
Escherichia
Bacteroides
Fusobacterium
Raoultella
Shigella
Blautia
Clostridium
Campylobacter
Megamonas
Flavonifractor
amin_aph3-I
amin_aph6-I
bac_bacA
beta_OXA-85
tet_tet37
tet_tetA
tet_tetQ
van_vanR
van_vanS
B
OC
Escherichia
Citrobacter
Shigella
Streptococcus
Plesiomonas
Clostridium
Enterobacillus
Pseudomonas
Duganella
Salmonella
bac_bacA
fosm_rosA
mul_acrA
mul_acrB
mul_emrB
mul_emrD
mul_mdtE
mul_mdtM
mul_mexX
mul_multidrug transporter
mul_omp36
mul_ompF
mul_ompR
unc_cobl-alamin
unc_transcriptional-cpxR
C
HE
Prevotella
Bacteroides
Clostridium
Alistipes
Hungatella
Bifidobacterium
Peptoclostridium
Ruminococcus
Erysipelatoclostridium
Blautia
bac_bacA
bac_bcrA
beta_CfxA2
chl_catB
mac_lsa
mac_vatB
mac_vatE
mul_multidrug_ABC_transporter
tet_tet32
tet_tet37
tet_tetQ
tet_tetracycline resistance protein
van_vanR
van_vanS
van_vanU

Figure S2 The distributions of ARG subtypes and their abundances in the total annotated ARGs subtypes in giant panda and red panda metagenomes (visualized by Circos(Krzywinski et al., 2009)). The length of the bars on the outer-ring represents the percentage of gut microbiome groups (genera level) in each ARGs subtype. Each gut microbiome genus was represented by a specific ribbon color, and the width of each ribbon demonstrates the abundance of each genus in the ARGs subtype. **A**. Qinling (wild Qinling population). **B**. Qionglai (wild Qionglai population). **C**, XXL (wild Xiaoxiangling population). **D**. Chengdu (captive Chengdu population). **E**. Yaan (captive Yaan population). **F**. RPxxl (wild red panda Xiaoxiangling population). amin, aminoglycoside. bac, bacitracin. beta, beta-lactam. ble, bleomycin. chl, chloramphenicol. fosm, fosmidomycin. kas, kasugamycin. mac, macrolide-lincosamide-streptogramin. mul, multidrug. poly, polymyxin. sul, sulfonamide. tet, tetracycline. tri, trimethoprim. unc, unclassified. van, vancomycin.

Bamboo-eating panda gut microbiome and their ARGs (subtype level)

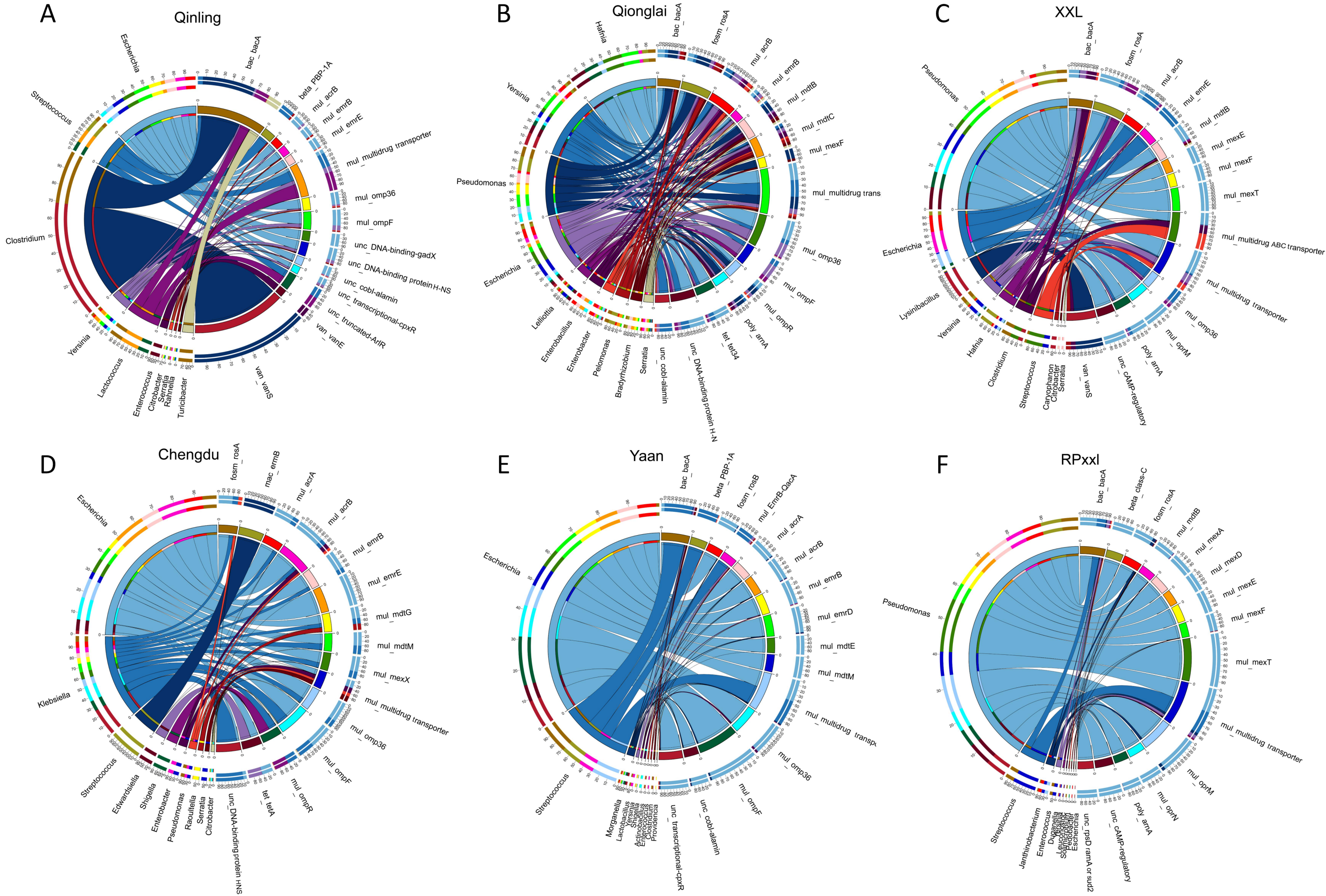

References:

Guo, W., Mishra, S., Wang, C., Zhang, H., Ning, R., Kong, F., Zeng, B., Zhao, J., and Li, Y. (2019). Comparative Study of Gut Microbiota in Wild and Captive Giant Pandas (Ailuropoda melanoleuca). *Genes* 10**,** 827.

Krzywinski, M., Schein, J.E., Birol, I., Connors, J.M., Gascoyne, R.D., Horsman, D., Jones, S.J.M., and Marra, M.A. (2009). Circos: An information aesthetic for comparative genomics. *Genome Research* 19**,** 1639-1645.

Wu, Q., Wang, X., Ding, Y., Hu, Y., Nie, Y., Wei, W., Ma, S., Yan, L., Zhu, L., and Wei, F. (2017). Seasonal variation in nutrient utilization shapes gut microbiome structure and function in wild giant pandas. *Proceedings of the Royal Society B: Biological Sciences* 284**,** 20170955.

Zhang, W., Liu, W., Hou, R., Zhang, L., Schmitz-Esser, S., Sun, H., Xie, J., Zhang, Y., Wang, C., and Li, L. (2018). Age-associated microbiome shows the giant panda lives on hemicelluloses, not on cellulose. *The ISME journal* 12**,** 1319-1328.

Zhu, L., Wu, Q., Deng, C., Zhang, M., Zhang, C., Chen, H., Lu, G., and Wei, F. (2018a). Adaptive evolution to a high purine and fat diet of carnivorans revealed by gut microbiomes and host genomes. *Environmental microbiology* 20**,** 1711-1722.

Zhu, L., Yang, Z., Yao, R., Xu, L., Chen, H., Gu, X., Wu, T., and Yang, X. (2018b). Potential mechanism of detoxification of cyanide compounds by gut microbiomes of bamboo-eating pandas. *MSphere* 3.
